# Role of exercise on ncRNAs and exosomal ncRNAs in preventing neurodegenerative diseases: a narrative review

**DOI:** 10.1186/s10020-025-01091-y

**Published:** 2025-02-07

**Authors:** Shangwu Liu, Runhong Zhang, Jamal Hallajzadeh

**Affiliations:** 1 Department of Physical Education, Lyuliang University, Lishi, 033000 Shanxi China; 2 Research Center for Evidence-Based Health Management, Maragheh University of Medical Sciences, Maragheh, Iran

**Keywords:** Exercise, Neurodegenerative diseases, Non-coding RNAs, Synaptic function, Cell signaling

## Abstract

Engaging in activity has proven to have beneficial effects on different facets of well-being, such as conditions related to the deterioration of the nervous system. Non-coding RNAs (ncRNAs) and exosomal ncRNAs associated with vesicles have been recognized as influencers of gene expression and cell signaling, potentially contributing to the positive impact of physical activity on neurodegenerative conditions. It is hypothesized that exercise-induced changes in ncRNA expression may regulate key processes involved in neuroprotection, including neuroinflammation, oxidative stress, protein aggregation, and synaptic function. Exercise has shown promise in preventing neurodegenerative diseases (NDs), and ncRNAs and exosomal ncRNAs are emerging as potential mediators of these benefits. In review, we explored how ncRNAs and exosomal ncRNAs play a role in enhancing the impacts of activity on neurodegenerative disorders for future treatments. Research studies, both preclinical and clinical, that have documented the use of various exercises and their effects on ncRNAs and exosomal ncRNAs for the treatment of NDs have been compiled and enlisted from the PubMed database, spanning the time period from the year 2000 up to the current time. Studies show that manipulating specific ncRNAs or harnessing exercise-induced changes in ncRNA expression and exosomal cargo could potentially be utilized as therapeutic strategies for preventing or treating NDs. In conclusion, studies suggest that various exercise modalities, including aerobic, resistance, and high-intensity interval training, can modulate the expression of ncRNAs and exosomal ncRNAs in the context of NDs. The altered ncRNA profiles may contribute to the neuroprotective and therapeutic effects observed with exercise interventions. However, more research is needed to fully understand the underlying mechanisms and to further explore the potential of exercise-induced ncRNA signatures as biomarkers and therapeutic targets for neurodegenerative disorders.

## Introduction

Neurological disorders encompass a wide range of conditions that affect both the central nervous system (CNS), including the brain and spinal cord, and the peripheral nervous system (Rittiner et al. 2020). These disorders significantly impact various aspects of health, including cognition, movement, sensory functions, and overall neurological well-being. Neurological disorders represent a significant proportion of the Global Disease Burden (GDB) (Huang et al. 2023). They often rank among the leading causes of disability-adjusted life years (DALYs) lost, surpassing many other disease categories (Huang et al. 2023). The prevalence and incidence of neurological diseases can differ markedly across geographical regions (Huang et al. 2023). Factors such as genetic predisposition, environmental exposures, and accessibility to healthcare services contribute to these variations (Huang et al. 2023). For instance, certain diseases, like Parkinson diseases (PD), exhibit higher prevalence rates in specific areas of the world (Huang et al. 2023). The implications of these disorders are profound, affecting both morbidity and mortality rates globally (Huang et al. 2023).

Recent research has indicated that engaging in physical exercise (PE) is associated with a reduced risk of developing neurodegenerative conditions and can enhance cognitive abilities. A meta-analysis has revealed that various PE programs can benefit individuals with dementia, leading to significant improvements in cognitive function, memory retention, and daily performance (Zhou et al. 2022). However, the underlying mechanisms that facilitate these benefits remain unclear.

One proposed mechanism is the modulation of ncRNAs by exercise, which may play a crucial role in neuroprotection. Among these, microRNAs (miRNAs) are particularly noteworthy as they regulate gene expression by binding to messenger RNAs (mRNAs), thereby influencing their translation or degradation. Altered expression profiles of miRNAs have been associated with several neurological diseases, affecting processes such as neuroinflammation, oxidative stress, and mitochondrial dysfunction (Catanesi et al. 2020).

Engaging in physical activity has been shown to modify miRNA levels in the brain, potentially yielding neuroprotective effects (Fischetti et al. 2023). For example, exercise can upregulate miRNAs that promote neuronal survival and synaptic plasticity, while also regulating neuroinflammation (Fischetti et al. 2023). Additionally, long non-coding RNAs (lncRNAs), which do not encode proteins but are involved in regulating gene expression, have also been implicated in neurodegenerative processes (Aliperti et al. 2021). Changes in lncRNA expression due to exercise can affect transcriptional regulation, chromatin remodeling, and neuronal development (Chen et al. 2024).

Understanding the specific ncRNAs involved in the pathogenesis of neurological diseases and their response to exercise may open new avenues for therapeutic interventions. This narrative review aims to explore the interplay between exercise, ncRNAs, and exosomal ncRNAs in the context of preventing neurodegenerative diseases. Although existing literature has recognized the benefits of exercise and the role of ncRNAs in neuroprotection, few reviews have integrated these aspects comprehensively. This review seeks to fill this critical gap by examining how exercise-induced changes in ncRNA expression influence mechanisms such as neuroinflammation and oxidative stress. Insights gained from this exploration may inform future therapeutic strategies that leverage exercise as a preventive or mitigative measure against neurological disorders.

## Biogenesis of ncRNAs

It’s clear that RNA molecules do much more than only function as a framework for the production of proteins. Non-coding transcripts are considered to be as adaptable as proteins, facilitating them to control a significant portion of biological activities, given that RNA can generate three-dimensional structures and interact with DNA, proteins, and other RNA molecules. Notably, during the past ten years, there has been a notable growth in the quantity and classification of these valuable ncRNAs, which differ in length from approximately twenty to thousands of nucleotides. The two main kinds of regulatory RNAs, small and lncRNAs, are recognized by their length, which is an essential element.

### MiRNAs biogenesis

**Fig. 1:**
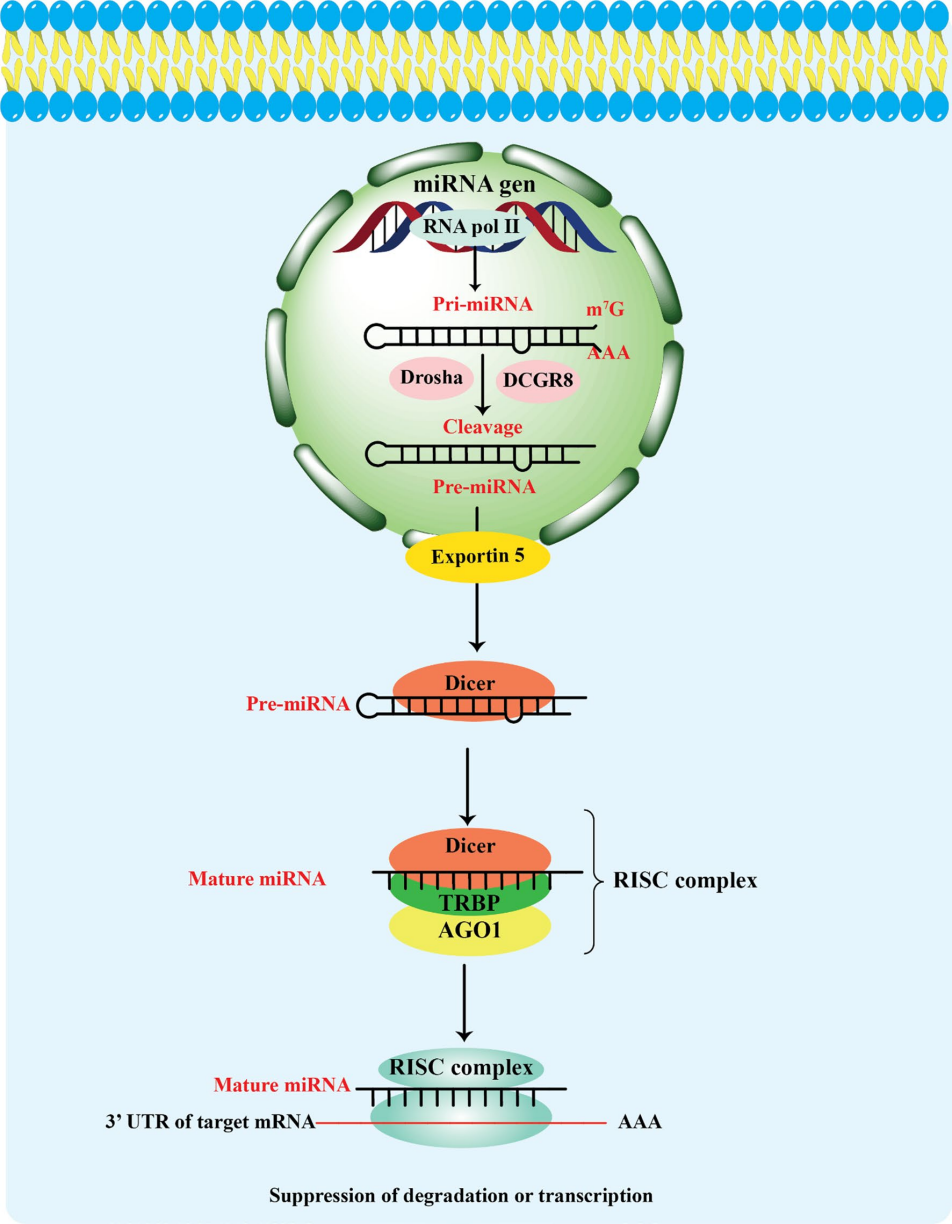
MiRNA biogenesis. RNA polymerase II (Pol II) transcribes miRNA genes to create primary transcripts (pri-miRNAs), which is a step in the miRNA biogenesis process. After processing the parent transcript, the Drosha-DiGeorge syndrome critical region gene 8 (DGCR8) complex produces around 70 nucleotide pre-miRNAs. Exportin 5 can identify these pre-miRNAs due to their short stem and 2-nt 3′ overhang. RNase III Dicer catalyzes the synthesis of miRNA duplexes after it has been exported. The pre-miRNA processing and RISC (RNA-induced silencing complex) assembly are mediated by Dicer, TRBP, and AGO 1–4. A single-stranded miRNA that is partly complementary to the target mRNA is produced when one strand of the miRNA duplex is deleted inside this complex. The 3′ UTR of mRNA targets is complementary to the “seed” sequence

RNA polymerase II transcribes the miRNA gene to start the process (O’Brien et al. 2018; Shang et al. 2023). These genes may be found in protein-coding gene’s introns or intergenic regions. The initial transcript, known as the pri-miRNA, is typically several hundred to thousands of nucleotides long and forms a hairpin structure. An enzyme called Drosha and its cofactor DGCR8 (DiGeorge Syndrome Critical Region 8) identify and cleave the pri-miRNA in the nucleus, to produce a precursor miRNA (pre-miRNA). The nuclear export factor Exportin-5 then moves the pre-miRNA from the nucleus to the cytoplasm by forming a complex with Ran-GTP. An enzyme known as Dicer further processes the pre-miRNA in the cytoplasm (O’Brien et al. 2018; Shang et al. 2023), along with its partner protein TRBP (TAR RNA-binding protein) (Shang et al. 2023). Dicer cleaves the pre-miRNA hairpin structure, generating a short double-stranded RNA molecule called the miRNA duplex. One strand of the miRNA duplex is chosen to be the mature miRNA, while the other strand, referred to as the miRNA* or passenger strand, is usually destroyed (O’Brien et al. 2018). The selection process is guided by the relative stability and composition of the duplex ends, as well as the asymmetry of thermodynamic properties. The mature miRNA strand associates with Argonaute (AGO) proteins within the RNA-induced silencing complex (RISC), forming the mature RISC complex (O’Brien et al. 2018; Shang et al. 2023). The RISC complex serves as the effector complex for miRNA-mediated gene regulation. Base pairing between the mature miRNA and complementary sequences in the target mRNA allows the mature miRNA within the RISC complex to direct the complex to particular target mRNAs. The level of complementarity between the miRNA and target mRNA determines whether this interaction results in translational repression or mRNA destruction (Fig.1) (O’Brien et al. 2018).

## LncRNAs biogenesis

lncRNAs have a significant impact on various aspects of human development and disease. They participate in vital processes such as the production of organs, tissue differentiation, and embryonic development (Herman et al. 2022; Winkle et al. 2021). Furthermore, chromatin structure, epigenetic changes, and gene expression are all regulated by lncRNAs. They are involved in immune response modulation, stress response, and cellular homeostasis maintenance (Herman et al. 2022; Winkle et al. 2021). Dysregulation of lncRNAs has also been connected to a number of human illnesses, such as immunological, cardiovascular, neurodegenerative, and cancer problems (Herman et al. 2022; Winkle et al. 2021). Understanding the functions and mechanisms of lncRNAs in these contexts is crucial for advancing our knowledge of human biology and exploring potential therapeutic interventions. The production of lncRNAs is highly dependent on the specific type of cell and its developmental stage (Mahalakshmi et al. 2020; Ilieva 2024). The biogenesis of lncRNAs is regulated by stimuli that are specific to the particular cell type and developmental stage (Mahalakshmi et al. 2020; Ilieva 2024).

**Fig. 2:**
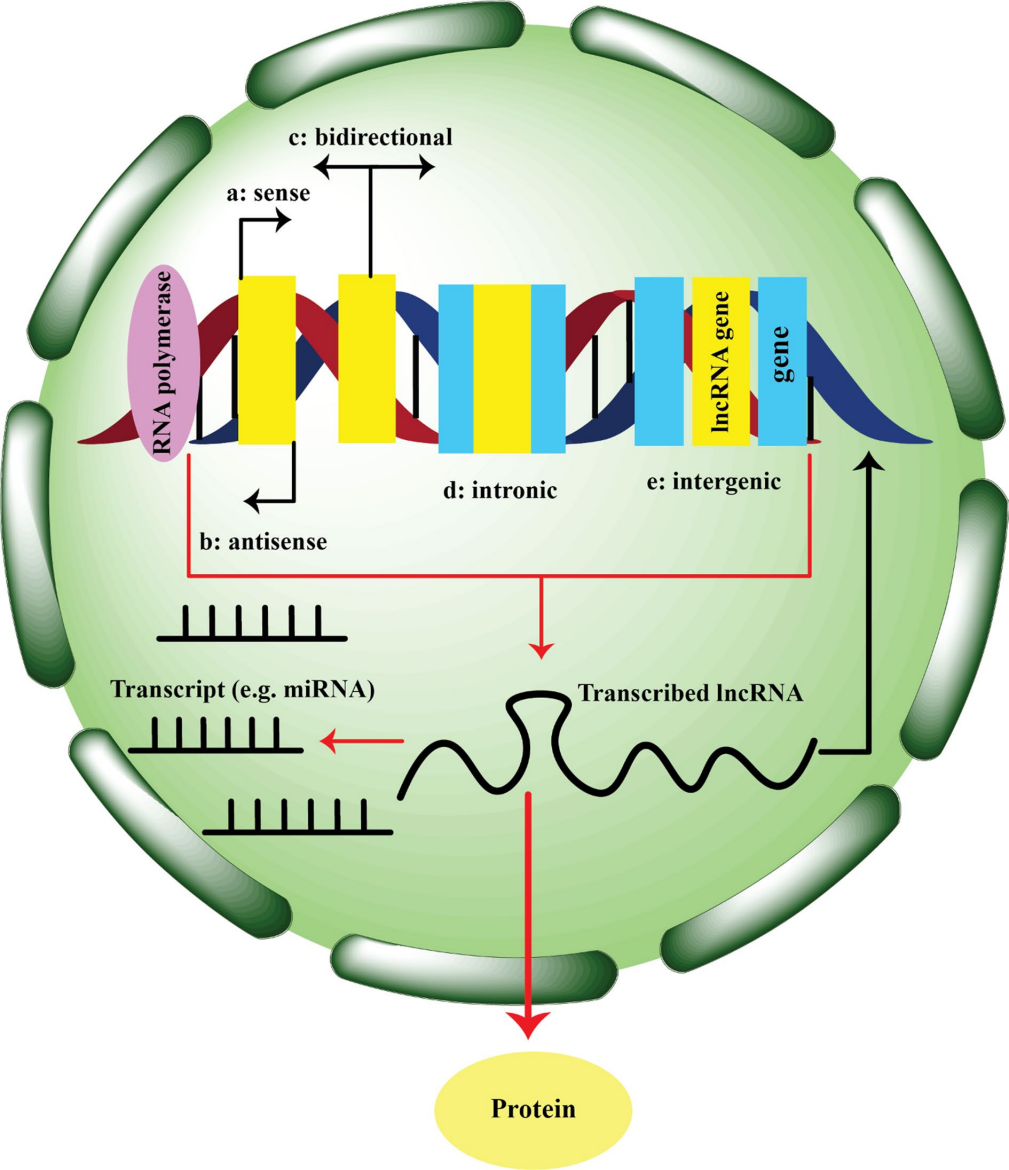
LncRNA biogenesis. LncRNAs are classified as sense, anti-sense, bidirectional, intronic, or intergenic lncRNAs based on where in the genome they are found. RNA polymerases transcribe them, and they are frequently polyadenylated, spliced, and 5′ capped. While some lncRNAs are translated into proteins, the majority of them work directly on the regulation of the genome or as binding partners for molecules that are either within (e.g., transcripts such as mirRNAs) or outside the nucleus (e.g., proteins)

Diverse DNA elements found in eukaryotic genomes are used to transcribe diverse kinds of lncRNAs. These consist of intergenic regions, enhancers, and promoters (Ayari et al. 2023). In addition to their role in gene regulation, enhancers can also give rise to lncRNA transcripts. These lncRNAs, known as enhancer-derived lncRNAs, can interact with target genes in cis or trans to modulate gene expression. Promoters are DNA sequences that initiate the transcription of genes. While most promoters are associated with protein-coding genes, some can also serve as the starting points for lncRNA transcription. These promoter-associated lncRNAs (plncRNAs) can regulate nearby genes or have independent functional roles. Intergenic lncRNAs are transcribed from intergenic regions and can have diverse regulatory functions, such as guiding chromatin remodeling complexes or modulating gene expression in trans (Ayari et al. 2023). The biogenesis of lncRNAs involves several mechanisms, including cleavage, capping, and circularization (Fig. 2). In some cases, lncRNAs are transcribed as longer precursor transcripts that undergo cleavage by the endonuclease RNase P (Jiang et al. 2022; Souza et al. 2022). RNase P recognizes specific RNA structural features, such as a conserved RNA motif or a specific secondary structure, and cleaves the precursor RNA to generate mature ends of the lncRNA (Jiang et al. 2022; Souza et al. 2022). Some lncRNAs acquire modified nucleotides and a protein complex known as a small nucleolar ribonucleoprotein (snoRNP) at their ends. These modifications and protein complexes act as caps that protect the lncRNA from degradation and facilitate its stability and processing. snoRNP complexes guide the modification of specific nucleotides within the lncRNA, which can influence its function and localization (Jiang et al. 2022; Souza et al. 2022). Back-splicing is the process that creates circular lncRNAs. In this process, the 3’ and 5’ ends of a linear RNA molecule are covalently joined to form a closed circular shape. Genes that bind to RNA and/or RNA secondary structures, such as complementary sequences or inverted repeats, are responsible for back-splicing. Circular lncRNAs have been shown to have distinct stability, localization, and regulatory functions compared to linear lncRNAs (Jiang et al. 2022; Souza et al. 2022). In recent studies, researchers have identified a distinct type of sub-nuclear structures called “paraspeckles” that form in the nucleus of mammalian cells. These paraspeckles are found in close association with particular lncRNAs called NEAT1 and NEAT2/MALAT1 during their biogenesis (Zhang et al. 2022). Through RNA interference (RNAi) experiments targeting 40 different paraspeckle proteins (PSPs), researchers have made an important discovery. They have identified four specific PSPs that play essential roles in the formation of paraspeckle (Oliver and Mandyam 2018; Penning et al. 2021). Although the mechanisms of synthesis and regulation of different lncRNAs are not yet fully understood, ongoing research in the next few years is expected to provide deeper insights into their biogenesis and functions.

## NcRNAs and neurodegenerative diseases

Cutting-edge scientific studies on ncRNAs have illuminated the molecular pathways that are fundamental factors in NDs. Abundant research has demonstrated that ncRNAs play a prominent role in a number of biological processes linked to the onset of neurodegenerative illnesses. Additionally, the common imbalances in NDs involve specific subtypes of ncRNAs, such as miRNAs, circular RNAs, and lncRNAs (Valizadeh et al. 2024).

MiRNAs have a role in modulating immune responses and maintaining immune balance by regulating genes involved in immune cell development, activation, proliferation, and differentiation (Readhead et al. 2020). They have the ability to affect immune cells, such as microglia in the central nervous system (CNS) (Estfanous et al. 2021; Li et al. 2019)and immune cells that infiltrate in neurodegenerative disorders. Furthermore, miRNAs regulate the upkeep and development of stem cells, including neural stem cells in the brain (Yang et al. 2017). Neural stem cell proliferation and differentiation into neurons and glial cells are significantly influenced by certain miRNAs, including the miR-17–92 cluster and miR-153 (Andersson et al. 2010; Qiao et al. 2020). Dysregulation of these miRNAs can disrupt the delicate equilibrium between self-renewal and differentiation, potentially contributing to neuronal loss observed in NDs. In addition to their roles in immune and stem cell regulation, miRNAs also have a significant impact on signal pathways associated with NDs. For example, the Wnt/β-catenin pathway, which is important in cell survival, proliferation, and differentiation, can be modulated by miRNAs (Zhang et al. 2022). Dysregulation of this pathway is linked to NDs, and miRNAs can directly target components of this pathway to influence neuronal differentiation, synaptic plasticity, and neuroinflammation (Zhang et al. 2022). Similarly, miRNAs can target other pathways such as the PI3K, mammalian target of rapamycin (mTOR), Notch, RelA/ApoE, MAPK, PTEN, NF-κB, and Nrf2 pathways, which are implicated in neurodegeneration (Bian et al. 2013; Qiao et al. 2020; Xing et al. 2020; Raffaele et al. 2023). Through their regulation of these pathways, miRNAs can impact various cellular processes relevant to NDs, including neuronal survival, synaptic function, neuroinflammation, and cell fate determination. Furthermore, recent evidence suggests that miRNAs may indirectly affect NDs through their interaction with the gut microbiota (Moloney et al. 2019; Lukiw 2020). Studies indicate that miRNAs and the gut microbiota mutually regulate each other, and this interaction can influence physiological and pathological processes via the miRNA brain-gut axis (Moloney et al. 2019; Lukiw 2020). Looking into this axis may lead to the discovery of new treatment targets for a range of ailments. Emerging evidence suggests that lncRNAs have significant contributions to the formation and operation of the nervous system. Apart from their role in adult NDs, lncRNAs also play crucial roles in neurodevelopment. They participate in processes such as neural stem cell maintenance, neuronal differentiation, and synapse formation. Dysregulation of lncRNAs during development can have long-lasting effects and may contribute to the predisposition or initiation of NDs later in life. LncRNAs exert their regulatory effects through diverse mechanisms. They can act as scaffolds, guiding protein complexes to specific genomic loci or interacting with DNA, RNA, or proteins to modulate gene expression. In addition, lncRNAs have the ability to function as competitive endogenous RNAs (ceRNAs), which sequester miRNAs and stop them from interacting with target mRNAs (Wang et al. 2020; Khorkova et al. 2015). Moreover, lncRNAs have the ability to control epigenetic changes, including methylation of DNA and changes to histones, which impacts the structure of chromatin and the expression of genes (Robinson et al. 2020). Several lncRNAs have been implicated in specific NDs. For example, beta-secretase 1(BACE1)-AS, BC200, and NEAT1 have been associated with Alzheimer’s disease (AD) (Modarresi et al. 2011; Lukiw et al. 1992). LincRNA-p21, SNHG1 and HOTAIR have been linked to Parkinson’s disease (PD) (Xu et al. 2018; Qian et al. 2019). HTT-AS, ABHD11-AS1, HAR1, TUNA, and brain-derived neurotrophic factor (BDNF)-AS have been implicated in Huntington’s disease (Riva et al. 2016; Lin et al. 2014). These disease-specific lncRNAs can directly modulate the expression of genes involved in disease pathogenesis or impact other regulatory processes (Riva et al. 2016). In addition to their roles in gene expression regulation, lncRNAs can influence RNA processing and transport (Herman et al. 2022). They can affect alternative splicing events, leading to the production of different protein isoforms. LncRNAs can also influence mRNA stability, localization, and translation, thereby modulating protein expression levels and cellular functions (Herman et al. 2022). Dysregulation of these processes can contribute to the pathogenesis of NDs (Qin et al. 2022). Targeting disease-associated lncRNAs holds therapeutic potential for NDs. Strategies such as antisense oligonucleotides, RNA interference, or small molecules can be employed to modulate the expression or function of specific lncRNAs (Winkle et al. 2021). Therapeutic interventions aimed at restoring normal lncRNA functions or reducing the expression of disease-associated lncRNAs could potentially mitigate disease progression or ameliorate symptoms.

## Physical exercise and neurodegenerative diseases

Physical exercise has demonstrated neuroprotective effects in neurodegenerative conditions, and one of the emerging areas of research focuses on the role of ncRNAs. These molecules, including miRNAs and lncRNAs, are critical regulators of gene expression and can influence various biological processes. Several mechanisms by which physical exercise may provide neuroprotective effects through the regulation of ncRNAs. Exercise can increase the levels of neurotrophic factors like BDNF, which are vital for the survival and adaptability of neurons (Mahalakshmi et al. 2020). NcRNAs regulate the transcription and stability of BDNF mRNA, thereby affecting its availability and role in neuroprotection (Ilieva 2024).

Physical activity can lessen neuroinflammation by adjusting the expression of pro-inflammatory cytokines (Ayari et al. 2023). NcRNAs significantly contribute to this process by targeting mRNAs that are part of inflammatory pathways, which may reduce the expression of inflammatory mediators in the brain (Jiang et al. 2022). Exercise can boost the brain’s antioxidant defenses (Souza et al. 2022). NcRNAs help regulate the expression of genes involved in oxidative stress responses, including those that code for antioxidant enzymes, thus protecting neurons from oxidative injury (Zhang et al. 2022). NcRNAs play a role in regulating neurogenesis (Oliver and Mandyam 2018). Changes in specific miRNAs and lncRNAs due to exercise may encourage the proliferation and differentiation of neural progenitor cells, aiding in the formation of new neurons in the hippocampus (Penning et al. 2021; , Liu et al. 2023). NcRNAs are essential for synaptic plasticity, which is crucial for learning and memory (Ge et al. 2023; Hu and Li 2017). Exercise can affect the expression of miRNAs that target synaptic proteins, potentially improving synaptic function and connectivity (Goldberg et al. 2021). Exercise can influence the expression of ncRNAs that regulate stress response pathways. By altering the levels of stress-related ncRNAs, exercise may protect neurons from damage caused by stress (Mahalakshmi et al. 2020). NcRNAs can enhance communication between neurons and glial cells (Marangon et al. 2022). Changes in ncRNA levels due to exercise can strengthen the supportive functions of glial cells, such as astrocytes and microglia, in preserving neuronal health (Marangon et al. 2022).

## Beneficial actions of physical exercise in Alzheimer’s disease

It has been demonstrated that regular exercise improves mental health, mood, and cognitive performance in AD patients (De la Rosa et al. 2020). It improves memory, attention, and executive functioning, promotes blood flow to the brain, and supports brain health and cognitive performance. Exercise also reduces symptoms of depression and anxiety, improves overall psychological well-being, and enhances quality of life. It triggers the brain’s natural mood-enhancing hormone, endorphin release (Mikkelsen et al. 2017). Regular exercise may slow down the progression of cognitive decline in AD, preserving cognitive abilities and delaying the onset of severe impairments (Qin et al. 2022). Additionally, it improves physical function by strengthening, extending, balancing, and coordinating muscles, all of which can help people maintain their independence and lower their chance of accidents and falls. Exercise programs focusing on aerobic conditioning, strength training, and balance exercises are particularly beneficial. Regular exercise supports activities of daily living, such as dressing, grooming, and mobility. It also has positive effects on cardiovascular health (Chen et al. 2022), especially for individuals with AD who may be at an increased risk of cardiovascular problems.

### Modulation of ncRNAs by physical exercise in Alzheimer’s disease

AD is a progressive neurodegenerative condition referred to as a neurodegenerative disease. It is characterized by a gradual deterioration in cognitive abilities and memory loss. Notable indicators of the disease include the presence of senile plaques, neurofibrillary tangles found within cells, and the accumulation of amyloid-beta (Aβ) outside cells. The specific causes and mechanisms behind the development of the disease are still largely unclear (Fernandez et al. 2019). In recent years, numerous ncRNAs have been shown to play important roles in the regulation of neuronal differentiation and death, contributing to the progression of neurological disorders (Tables 1, 2) (Qin et al. 2022; Ansari et al. 2019; Lu et al. 2022). Regular physical activity has been found to have positive impacts on brain structure and cognitive function (Vesperman et al. 2018; Kelly 2018). Research has shown that engaging in physical activities can lead to beneficial changes in brain structure, such as increased volume in regions related to memory and learning (Vesperman et al. 2018). Physical activity also promotes neuroplasticity, which helps the brain form new connections and supports processes like memory and learning. Exercise has been associated with improvements in executive functioning, working memory, attention, and processing speed in terms of cognitive function (Qin et al. 2022; Vesperman et al. 2018; Panza et al. 2018; Cui et al. 2018). Regular exercise can also improve mental and emotional well-being, which in turn benefits cognitive performance. Exercise’s impact on vascular health and its potential neuroprotective effects further contribute to its benefits for cognitive function. It is noteworthy that the specific type, intensity level, and duration of PE may influence the outcomes. It has been demonstrated that both weight training and aerobic exercise are advantageous. However, further research is needed to fully understand the underlying processes that link regular PE to favorable impacts on brain structure and cognitive performance, despite the support from available data. While the release of growth factors like BDNF is believed to play a role in neuroplasticity and cognitive enhancement, the precise molecular and cellular mechanisms by which exercise influences brain structure and function are not fully elucidated. A study conducted by Lu et al. found that exercise has positive effects on cognitive recovery (Lu et al. 2022). Exercises have been shown to reduce escape latency, decrease distance traveled, increase the time spent in the target region, and prolong platform crossing durations. These findings suggest that exercise can improve cognitive function and performance in tasks related to spatial memory and navigation (Lu et al. 2022). Additionally, the study found that exercise had an inhibitory effect on inflammatory cytokines in mice. This suggests that physical activity can help reduce inflammation in the body, which is known to be associated with various health conditions, including cognitive decline. By inhibiting inflammatory cytokines, exercise may contribute to a healthier inflammatory response and potentially protect against cognitive impairments associated with inflammation (Du et al. 2018; Pang et al. 2019).

**Table 1:** Studies examining the effect of physical exercise interventions on ncRNAs expression in neurological disorders

Authors	Diseases	Exercise protocols	Model (in Vivo, human)	Sample type	Non-coding RNAs	Outcomes after physical exercise	Refs.
Qin et al. 2022	AD	Training on a bicycle for three months at 70% of maximal heart rate	Human	Blood	MiR-192-5p	When compared to AD sedentary individuals, the exercise group’s blood levels of miR-192-5p were lower.	Qin et al. (2022)
Li et al. 2020	AD	Training on a bicycle for three months at 70% of maximal heart rate	Human	Blood	MiR-129-5p	AD patients who exercised had greater blood levels of hsa-miR-129-5p than AD patients who did not exercise.	Li et al. (2020)
Lu et al. 2022	AD	Eight weeks, seven days a week, voluntary wheel running	Eight-month-old mice with double transgenic APP/PS1	Tissue	mmu-miR-130a-3p, HOTAIR	APP/PS1 mice had higher mmu-miR-130a-3p expression while VE animals had lower HOTAIR expression than the control group.	Lu et al. (2022)
Lu et al. 2022	AD	Three-month exercise intervention	Human	Blood	HOTAIR	After exercise, the level of HOTAIR was lower in 43 patients with AD compared to their HOTAIR level before exercise.	Lu et al. (2022)
Qin et al. 2022	AD	A four-week voluntary wheel running	Eight-month-old mice with double transgenic APP/PS1	Tissue	mmu-miR-192-5p	APP/PS1 animals in the voluntary exercise group had lower mmu-miR-192-5p than the group of sedentary mice.	Qin et al. (2022)
Li et al. 2020	AD	A four-week voluntary wheel running	Eight-month-old mice with double transgenic APP/PS1	Tissue	mmu-miR-129-5p	APP/PS1 animals in the voluntary exercise group had greater levels of mmu-miR-129-5p than the group of sedentary mice.	Li et al. (2020)
Shvarts-Serebro et al. 2021	AD	EE for eight weeks on RWs	7-month-old female mice, both WT and 5xFAD	Tissue	MiR-128	MiR-128 expression was downregulated	Shvarts-Serebro et al. (2021)
Dungan et al. 2020	AD	For twenty weeks, gradually increasing the weighted wheel running (PoWer)	3xTg-AD mice and 2-month-old WT female mice	Tissue	mmu-miR-132, mmu-miR-98, mmu-miR-130a, mmu-miR-29b, mmu-miR-15b-5p, mmu-miR-107, mmu-miR-29a, mmu-miR-129, mmu-miR-328, mmu-miR-140, mmu-miR-148b, and mmu-miR-29c	When comparing the 3xTg-AD mice in the exercise group to the sedentary mice group, mmu-miR-132 and mmu-miR-98 were lower while mmu-miR-130a, mmu-miR-29b, mmu-miR-15b-5p, mmu-miR-107, mmu-miR-29a, mmu-miR-129, mmu-miR-328, mmu-miR-140, mmu-miR-148b, and mmu-miR-29c were higher.	Dungan et al. (2020)
Dong et al. 2018	AD	Four weeks of voluntary wheel running	6-month-old SAMR1-trained WT males and SAMP8 mice	Tissue	mmu-miR-132	When compared to the sedentary mice group, the SAMP8 mice in the exercise group had reduced mmu-miR-132.	Dong et al. (2018)
Cosín-Tomás et al. 2014	AD	Eight weeks, seven days a week, voluntary wheel running	Mice SAMR1 and SAMP8 8-month-old WT females	Tissue	mmu-miR-98-5p, mmu-miR-15b-5p, mmu-miR-7a-5p, mmu-miR-148b-3p, mmu-miR-28-5p, mmu-miR-133b-3p and mmu-miR-105	In comparison to the sedentary mice group, the SAMP8 mice in the exercise group showed Greater levels of mmu-miR-98-5p, mmu-miR-15b-5p, mmu-miR-7a-5p, mmu-miR-148b-3p, and mmu-miR-28-5p, while mmu-miR-133b-3p and mmu-miR-105 were lower.	Cosín-Tomás et al. (2014)
Liu et al. 2019	PD	Regular aerobic exercise for 8 weeks	A PD model in rats	Tissue	MiR-3557, miR-324	The exercise group showed an upregulation of miR-3557 and a downregulation of miR-324 in comparison to the sedentary group.	Liu et al. (2019)
Da Silva et al. 2021	PD	An eight-week interval training program using a cycle ergometer for thirty minutes three times a week.	Human	Blood	MiR-103a-3p, miR-29a-3p, miR-106a-5	Following PE, the expression levels of miR-103a-3p, miR-29a-3p and miR-106a-5prose in the experimental group.	Da Silva et al. (2021)
Lohrasbi et al. 2022	MS	Five days a week for five weeks, a rat- RW with 100 mg/kg of royal jelly	A MS model in rats	Blood	MiR-34a-5p, miR155-3p	There was a significant drop in the expression levels of miR-34a-5p and miR155-3p in rats with MS.	Lohrasbi et al. (2022)
Yousefi Saqqezi et al. 2021	MS	For eight weeks, there will be four supervised workouts each week—three aerobic and one resistance.	Women with relapsing and remitting MS	Blood	MiR-326 and miR-155, miR-23b	MiR-326 and miR-155 expression increased in both groups but reduced in the exercise training group, miR-23b expression dropped in both the exercise training and control groups.	Yousefi Saqqezi et al. (2021)
Bao et al. 2014	TBI	Spontaneous RW exercise	A TBI mouse model	Tissue	MiR-34a, miR-21	In particular, miR-34a and miR-21 were linked to the healing process.	Bao et al. (2014)
Miao et al. 2015	TBI	RW for three weeks	A TBI mouse model	Tissue	MiR-874, miR-21, miR-92a, miR-124, let-7c, miR-138.	Prior to TBI, exercise increased the expression of miR-874, miR-21and miR-92a while downregulating the expression of miR-124, let-7c, and miR-138.	Miao et al. (2015)
Hu et al. 2015	TBI	For a duration of two weeks, the RWs were freely accessible to the mice in the groups of TBI-runners and sham-runners.	A TBI mouse model	Tissue	MiR-21	Spontaneous RW exercise decreased the elevated miR-21 levels in the hippocampus of TBI mice	Hu et al. (2015)
Wu et al. 2022	SCI	Two weeks of treadmill training (TMT), beginning one week following SCI	A SCI rat model	Tissue	Lnc MSTRG.12598.1, lnc MSTRG.18736.1	Exercise increased MSTRG.12598.1 and MSTRG.18736.1 levels	Wu et al. (2022)
Liu et al. 2012	SCI	Rats in the trial were given two 30-minute passive cycling sessions five days after SCI and again five days a week until the completion of the experiment. Twenty days of exercise were given to the rats in the Tx + Ex 10d and Tx + Ex + Rap groups.	A SCI rat model	Tissue	MiR-21, miR-199a-3p	MiR-21 expression was upregulated while miR-199a-3p expression was downregulated during exercise.	Liu et al. (2012)
Liu et al. 2010	SCI	The Tx + Ex 10d group of animals had 20 days of continuous, five-day exercise following spinal cord transection, including a 10-minute rest interval, a 45-rpm pedal rate, and complete range of motion.	A SCI rat model	Tissue	Let-7a, miR16, miR-21, miR15b	Exercise did not affect Let-7a or miR16, however it did dramatically raise miR-21 and reduce miR15b expression. After injury, an extended exercise program had no effect on miR expression in the Tx31d group.	Liu et al. (2010)

**Table 2:** Effects of exercise on exosomal ncRNAs in neurological diseases

Authors	Diseases	Exercise protocols	Type of models	Sample type	Non-coding RNAs	Expression	Refs.
Chen et al. 2022	Alzheimer’s disease	Exercise	Human	Blood	miR-215-5p	Up	Chen et al. (2022)
Liang et al. 2023	Alzheimer’s disease	Long-term exercise	5XFAD mice	Tissue	miR-532-5p	Up	Liang et al. (2023)
Liu et al. 2024	Alzheimer’s disease	Consistent exercise	Human	Blood	miRNA-483-5p, miRNA-502-5p	Up	Liu et al. (2024)

## The role of lncRNA by physical exercise in Alzheimer’s disease

HOTAIR (HOXD Transcript Antisense Intergenic RNA) is a lncRNA originally identified as being critical in regulating HOX gene clusters, which are essential for developmental processes. HOTAIR plays a crucial role in various biological processes, including chromatin remodeling and gene silencing. Elevated levels of HOTAIR have been linked to neurodegenerative disorders, including AD (Lu et al. 2022), where it may influence pathways associated with neuroinflammation and neuronal apoptosis. Some studies have suggested that HOTAIR may be involved in the regulation of genes associated with neurodegeneration and cognitive function. Combining the Position Weight Matrix-based Enrichment (PoWeR) technique with studies on HOTAIR enhances our understanding of gene regulation in Alzheimer’s Disease. This integrative approach can provide valuable insights into the mechanisms of neurodegeneration and identify potential avenues for therapeutic intervention (Lu et al. 2022). In contrast to the beneficial effects of PE in AD, a study has suggested that the overexpression of HOTAIR may have a detrimental impact on the recovery of spatial exploration in individuals with AD (Lu et al. 2022). This suggests that HOTAIR might not be beneficial in the context of AD. Research has shown that HOTAIR can indirectly influence cognitive ability and inflammation through its interactions with miR-130a-3p. Specifically, HOTAIR has been found to function as a competitive ceRNA by sponging miR-130a-3p, preventing miR-130a-3p from binding to its target mRNAs (Lu et al. 2022). By sequestering miR-130a-3p, HOTAIR can indirectly regulate the expression of genes targeted by miR-130a-3p. This may result in dysregulation of specific genes involved in cognitive function and inflammation, ultimately impacting cognitive ability and inflammatory processes.

It is still unclear how exactly HOTAIR affects AD and the specific mechanisms involved. However, it is speculated that dysregulation of HOTAIR may contribute to the pathogenesis and progression of AD by influencing gene expression and epigenetic modifications. The detrimental impact of HOTAIR overexpression on spatial exploration recovery further emphasizes the potential significance of understanding the role of HOTAIR in AD. This suggests that HOTAIR could play a role in the cognitive deficits associated with AD and may be a target for treatment approaches. In a separate study, Lu et al. (2022)discovered that individuals with AD expressed higher levels of HOTAIR compared to those without AD. Furthermore, lower Mini-Mental State Examination (MMSE) scores and higher ADAS-Cog scores, which are commonly used markers of cognitive impairment in AD, demonstrated that higher levels of HOTAIR expression were associated with worse cognitive performance (Lu et al. 2022). Interestingly, the study also found that a three-month exercise program improved cognitive performance in AD patients and reduced their relative serum expression of HOTAIR. This suggests that exercise may have a modulating effect on the development of AD by regulating the expression of HOTAIR (Lu et al. 2022). These findings suggest that exercise could potentially influence HOTAIR expression and contribute to the improvement of cognitive function in individuals with AD. By reducing HOTAIR expression, exercise may help mitigate the detrimental effects of HOTAIR on cognitive impairment.

## The role of miRNA by PE in Alzheimer’s disease

The pathophysiology of AD has been linked to dysregulation of miRNAs, which has been identified as a significant element in the disease’s progression. Changes in miR-132 expression have been identified in various neurological conditions, including AD (Lu et al. 2022). In individuals with AD, miR-132 levels have been found to be reduced, potentially contributing to the cognitive impairments associated with the disease. Animal studies have demonstrated that manipulating miR-132 levels can affect cognitive function and synaptic plasticity (Lu et al. 2022; Wang et al. 2017). The effects of voluntary aerobic PE were examined in an experimental investigation using the Senescence Accelerated Mouse-Prone 8 (SAMP8) mouse model of AD. The study revealed that untreated SAMP8 mice exhibited elevated levels of miR-132 in the hippocampus (Lu et al. 2022). However, when these mice engaged in voluntary aerobic PE, the expression of miR-132 was downregulated (Lu et al. 2022). Furthermore, the study observed that PE was effective in reducing the accumulation of amyloid precursor protein (APP) in the hippocampus (Lu et al. 2022). APP accumulation is associated with AD pathology, and inhibiting it contributed to the prevention of hippocampal degeneration. Consequently, the SAMP8 mice that underwent PE showed improvement in cognitive function.

In various AD models, preclinical research consistently demonstrates a decrease in miR-132 expression. This reduction has been observed not only in animal models but also in in vitro models and in AD patients (Hernandez-Rapp et al. 2016; Wang et al. 2017). The dysregulation of miR-132 in AD suggests its potential involvement in the disease’s development. The precise mechanisms by which miR-132 downregulation affects cognition and contributes to AD pathology are still under investigation. However, it is believed that miR-132 controls the expression of genes related to neuroinflammation, amyloid-β metabolism, and synaptic plasticity, all of which are crucial processes in AD (Hernandez-Rapp et al. 2016; Wang et al. 2017). Evidence suggests that miR-132 can be upregulated in the brain following PE, including swimming training and voluntary exercise. Numerous studies have examined how exercise influences the expression of miR-132 in both human and animal subjects. In animal studies, swimming training and voluntary exercise have been shown to increase miR-132 expression in the hippocampus (Habibi et al. 2017; Dong et al. 2018), a brain region important for learning and memory. This upregulation of miR-132 has been associated with enhanced neuroplasticity and improved cognitive performance in various behavioral tasks (Habibi et al. 2017; Dong et al. 2018). Similarly, in human studies, exercise-induced upregulation of miR-132 has been observed (Radom-Aizik et al. 2012; de Gonzalo-Calvo et al. 2018). One study looked at the effects of various doses of acute aerobic exercise on blood samples of middle-aged male athletes before and after 10-kilometer, half-marathon, and marathon races and found an increase in miR-132 expression (de Gonzalo-Calvo et al. 2015). Another study investigated miRNA expression profiles in peripheral blood mononuclear cells (PBMCs) of young males before and after acute aerobic exercise and observed an upregulation of miR-132 in PBMCs following exercise (Radom-Aizik et al. 2012).

Several studies have reported altered expression of miR-15b in the brains and peripheral tissues of individuals with AD. In some cases, miR-15b levels were found to be decreased (Li and Wang 2018), while others observed increased expression. These alterations suggest that miR-15b could be involved in the molecular mechanisms underlying AD (Cosín-Tomás et al. 2014). It has been demonstrated that miR-15b controls the expression of genes related to amyloid-beta metabolism, a hallmark of AD (Li and Wang 2018; Gong et al. 2017). Research indicates that miR-15b, which targets genes including BACE1 and APP, can regulate the generation and clearance of amyloid-beta (Li and Wang 2018; Gong et al. 2017). Additionally, miR-15b has been linked to the control of neuronal survival and function by targeting genes involved in neuroprotective pathways, such as BDNF, which is essential for neuronal survival and plasticity. Dysregulation of miR-15b may disrupt these pathways and contribute to neurodegeneration in AD. Exercise-induced upregulation of miR-15b could potentially modulate Aβ metabolism and contribute to the clearance of Aβ plaques in the brain (Cosín-Tomás et al. 2014). Research has demonstrated that the expression of miR-29 is altered in AD, with some results indicating upregulation and others downregulation depending on the specific brain area and stage of the disease. One of the key functions of miR-29 in AD is its regulatory role in the expression of certain genes involved in Aβ processing. It has been shown that miR-29 targets the BACE1 gene, an enzyme involved in the synthesis of Aβ (Hébert et al. 2008; Zong et al. 2011). Dysregulation of miR-29 may lead to increased BACE1 expression and subsequent accumulation of Aβ plaques, which are a hallmark of AD. Additionally, the modulation of Dicer expression may influence the processing and activity of miRNAs, including miR-29 (Shukla et al. 2011), which can further affect BACE1 expression and Aβ production (Dungan et al. 2020). Given that training for resistance and endurance raises the expression of BACE1 and Dicer (Zhang et al. 2018; Garner et al. 2020). In the context of 3xTg-AD mice, which harbor human transgenes associated with familial AD mutations, the expression of miR-29, BACE1, and Dicer has been examined in response to exercise training in the hippocampus (Dungan et al. 2020). The study found that the PoWeR exercise program (Dungan et al. 2019)resulted in muscle hypertrophy and increased myonuclear abundance in the mice. Furthermore, the hippocampal regions of 3xTg-AD mice showed increased expression of the Dicer gene, which is implicated in miRNA processing, after undergoing exercise training (Dungan et al. 2020). Compared to wild-type mice, sedentary 3xTg-AD animals exhibited significantly reduced levels of miR-29 (Dungan et al. 2020). However, after the PoWeR exercise program, the expression of miR-29 was elevated in the 3xTg-AD mice. This increase in miR-29 expression was accompanied by lower BACE1 gene expression and reduced levels of detergent-soluble Aβ1–42, a form of Aβ associated with AD pathology (Dungan et al. 2020). These results indicate that the PoWeR exercise program enhances the expression of the Dicer gene, which in turn modifies the expression of miRNAs in the brain. These changes in miRNA expression, such as the increased levels of miR-29, may contribute to a reduction in Aβ accumulation and potentially delay the progression of AD.

MiR-192-5p is recognized as a versatile regulatory factor and is thought to have a significant involvement in the pathological processes of various diseases (Qin et al. 2022; Raheja et al. 2018; Kang et al. 2019). Several studies have reported dysregulation of hsa-miR-192-5p in AD (Qin et al. 2022). In comparison to healthy controls, Qin et al.‘s study (Qin et al. 2022)revealed that brain tissue, cerebrospinal fluid, or blood samples from AD patients had different amounts of hsa-miR-192-5p expression (Qin et al. 2022). The possible involvement of hsa-miR-192-5p in the pathogenic processes of the illness is suggested by its dysregulation. According to this study, AD patients’ blood levels of miR-192-5p decreased following three months of exercise. Additionally, a negative link was shown between AD patients’ cognitive ability and their levels of miR-192-5p (Qin et al. 2022). In instance, it has been demonstrated that exercise increases the expression of miR-192-5p in the brain, especially in areas like the hippocampus that are linked to cognitive performance (Qin et al. 2022). Exercise-induced overexpression of miR-192-5p may be a factor in these people’s improved cognitive function. The specific mechanisms by which miR-192-5p may mediate cognitive improvement in AD are still being investigated. Nevertheless, it has been proposed that miR-192-5p may target genes related to synaptic plasticity, neuronal survival, and neuroinflammation—all of which are critical for cognitive function (Qin et al. 2022). Exercise-induced upregulation of miR-192-5p may modulate these pathways, leading to cognitive improvements in AD.

According to studies, miR-129-5p helps to reduce neuroinflammation in the spinal cord after ischemia-reperfusion damage (Li et al. 2021). Few research has examined the involvement of miR-129-5p in the development of AD, despite its potential significance. In the study conducted by Li et al. (2020), AD mice and patients underwent PE for a certain period of time. The researchers assessed neuroinflammation and cognitive function in addition to measuring the expression of miR-129-5p. The expression of miR-129-5p was shown to be elevated by PE in both AD mice and people (Li et al. 2020). Through the manipulation of miR-129-5p expression in the mice, the researchers found that knocking down miR-129-5p reversed the benefits of PE-induced enhanced cognitive performance and decreased inflammatory responses. Serum levels of miR-129-5p were shown to be linked with proinflammatory cytokines and indicators of cognitive function in AD patients (Li et al. 2020). A sizable cohort of AD-affected human brains and a control group were included in the Lau et al. (2013)investigation. In this study, the researchers created a profile of miRNAs based on their analysis of these samples (Lau et al. 2013). Among the noteworthy miRNAs found in the hippocampus of AD patients, miR-128 is the second most substantially changed miRNA, according to this study. Barak et al. also observed a significant decrease in the expression of this particular miRNA, miR-128, after exposing C57BL/6J mice to an enriched environment (EE) (Barak et al. 2013). MiR-128 is a highly abundant and enriched miRNA in the adult brains of both humans and mice (Lanza et al. 2023). It exhibits the highest expression levels in the human brain compared to other tissues (Lanza et al. 2023). The widespread expression of miR-128 across various brain regions indicates its significant involvement in processes that are shared among different types of neurons. There are multiple studies that link miR-128 to the functioning and plasticity of neurons (Lanza et al. 2023). Another study demonstrated the essential role of miR-128 in fear extinction learning, as it regulates several genes associated with neurotransmitter receptors, synaptic vesicle trafficking, and synapse formation (Ching and Ahmad-Annuar 2015). According to Shvarts-Serebro et al.‘s study (2021), miR-128 regulates the expression of two essential proteins involved in synaptic transmission: SNAP-25 and synaptotagmin1 (Syt1). Notably, miR-128 expression was discovered to be downregulated in a mouse model of AD termed 5xFAD, which is comparable to the change seen in the hippocampi of people with AD. It’s interesting to note that miR-128 expression levels dropped in normal mice that were exposed to an EE (Barak et al. 2013; Shvarts-Serebro et al. 2021). It is crucial to stress that there is a fundamental difference between the impact of EE and AD settings on synaptic plasticity and neuronal functioning. Additionally, the study showed that decreased expression of miR-128 in primary hippocampal cells from 5xFAD animals resulted in increased excitability and neural network activity.

### Beneficial actions of physical exercise in Parkinson’s disease

People with PD can benefit from regular exercise in a number of ways. It improves motor function by enhancing muscle strength, flexibility, balance, and coordination, which are often impacted by the disease (Motl 2020). Exercise programs that focus on aerobic conditioning, strength training, and specific movements can lead to improved motor control and mobility (Motl 2020). PE also enhances mobility and balance in individuals with PD. It improves walking ability, gait patterns, and postural stability. Incorporating activities such as walking, cycling, dancing, and tai chi into exercise programs can be particularly beneficial in enhancing these aspects. Exercise is beneficial in the management of PD motor symptoms, including stiffness, tremors, and bradykinesia, or sluggish movement (Motl 2020). Frequent exercise can enhance general motor function and lessen the severity of these symptoms. Cardiovascular issues are more common in people with PD. Frequent cardiovascular exercise reduces the risk of cardiovascular illnesses, increases cardiovascular fitness, and promotes general heart health (Motl 2020). Examples of this type of exercise include brisk walking, swimming, and cycling. Exercise has positive effects on mood and mental well-being in individuals with PD (Motl 2020). It reduces symptoms of depression and anxiety, improves psychological well-being, and enhances quality of life (Motl 2020). Endorphins are naturally occurring substances in the brain that enhance mood when they are released in response to PE. Emerging evidence suggests that exercise may have neuroprotective effects in PD (Motl 2020). PE promotes the production of neurotrophic factors, which support the survival and growth of neurons (Motl 2020). It may also help reduce oxidative stress and inflammation, which are implicated in the progression of PD. Regular exercise improves functional independence and supports activities of daily living in individuals with PD (Motl 2020). It assists with tasks such as dressing, grooming, and self-care by improving strength, flexibility, and motor coordination (Motl 2020).

### Modulation of ncRNAs by physical exercise in Parkinson’s disease

PD is a neurological illness that mostly impacts the motor system. PD is the second most common neurodegenerative disease, with an estimated lifetime risk of 2%. The substantia nigra, a part of the brain involved in motor control, gradually loses dopamine-producing cells, which is how it is identified. Dyskinetic symptoms, which include bradykinesia, tremors, stiffness, and postural instability, are brought on by a deficiency of dopamine. PD can also result in non-motor symptoms such as cognitive decline, anxiety, sadness, and sleep difficulties, as well as autonomic dysfunction. Although the precise etiology of Parkinson’s disease is still unknown, it is thought to be a result of a hereditary and environmental cocktail.

### The role of miRNA by physical exercise in Parkinson’s disease

Recent developments in our knowledge of the molecular mechanisms underlying PD have demonstrated the role of miRNAs in the disruption and dysregulation of systems linked to the control of gene expression levels implicated in the development of PD. Research indicates that miRNAs may have significance as a possible biological mediator of exercise-induced adaptation in both healthy individuals and those suffering from neurological disorders like PD (Da Silva et al. 2021). Using a rat model, the authors looked at how regular aerobic exercise affected PD. The researchers aimed to understand how regular exercise could impact the development and progression of PD symptoms. Two groups of rats were used in the study: one for aerobic exercise and the other for sedentary control. After eight weeks of consistent aerobic exercise, the aerobic exercise group was put through a 6-hydroxydopamine (6-OHDA)-induced PD lesion model. Prior aerobic activity strengthened the rats’ tolerance to the PD-inducing effects of 6-OHDA, the researchers found. In particular, the exercise group’s rats displayed decreased rotating behavior—a hallmark of PD—and enhanced damage resistance. The two groups’ rat brains were examined under a microscope, and the results showed clear differences. When compared to the sedentary group, the neurons, axons, and villi in the striatum—a part of the brain damaged by PD—were more organized and seemed bigger in the aerobic exercise group (Liu et al. 2019). These results imply that frequent aerobic exercise may preserve the brain areas damaged by PD (Liu et al. 2019). Additionally, they discovered that the aerobic exercise group had downregulation of miR-324 and overexpression of miR-3557 (Liu et al. 2019). These miRNAs were found to be closely associated with the calcium-modulating signaling pathway, which is involved in PD progression. Furthermore, the group that engaged in aerobic exercise demonstrated an increase in the expression of Ca/calmodulin-dependent protein kinase II (CaMK2α) and activation of the proteins involved in the PI3K/ mTOR pathway (Liu et al. 2019). These changes in protein expression suggest that regular aerobic exercise may modulate the calcium signaling pathway, potentially contributing to the protective effects against PD.

Research has shown interest in the connection between miRNAs, PD, and cognitive function (Table 1). One prevalent non-motor symptom of PD is cognitive impairment, which impacts executive function, memory, attention, and other cognitive domains. According to studies, miRNAs may affect the expression of genes related to synaptic plasticity and neuronal function, which may lead to the cognitive impairment seen in PD. In the pathophysiology of PD, deregulation of miRNAs, such as miR-29a-3p, miR-103a-3p, and miR-106a-5p, has been linked (Da Silva et al. 2021; Serafin et al. 2015). Serafin et al. ( 2015)examined the miR-29a-3p and miR-103a-3p expression levels in PBMCs from PD patients. According to the study, PD patients had dysregulated expression of miR-103a-3p and miR-29a-3p, which is abnormal when compared to healthy people (Serafin et al. 2015). Furthermore, the overexpression of these miRNAs was observed specifically in PBMCs of PD patients who were treated with L-dopa, a common medication used for symptom management in PD (Serafin et al. 2015). Significant biological processes connected to motor function and the development of the CNS have been linked to these miRNAs. Research has indicated that miR-106a-5p regulates the expression of autophagy-related gene 7, and that the loss of dopaminergic neurons and accumulation of α-synuclein are linked to the suppression of this gene (Hao et al. 2017; Ahmed et al. 2012). It has been discovered that miR-103a-3p is connected to the Wnt signaling pathway. Dysregulation of this system, which is linked to dopaminergic neuron survival, may play a role in PD progression. G protein-coupled receptor 37 (GPR37) has been identified as one of the targets of miR-29a-3p. The excessive accumulation of GPR37 within cells is known to be neurotoxic and has been linked to PD. Relevant motor and non-motor characteristics for PD have been seen when GPR37 is inactivated or disrupted.

Research has shown that exercise can have positive effects on cognitive function in individuals with PD. Exercise promotes neuroplasticity and the growth of new neurons, which can help maintain cognitive function. It has been discovered that exercise raises BDNF levels, a protein that is essential for supporting neuronal survival, development, and differentiation. The capacity of synapses to become stronger or weaker over time is known as synaptic plasticity, and BDNF is known to improve it (Müller et al. 2020). Additionally, it shields already-existing neurons from harm or deterioration and encourages the development of new synapses. Conversely, exercise promotes the growth of new blood vessels. Angiogenesis can promote neuron survival and proliferation as well as improve brain function (Hötting and Röder 2013). Furthermore, research has been done on the function of miRNAs in modulating the association between exercise and cognitive performance in PD (Da Silva et al. 2021). Exercise-induced changes in miRNA expression may contribute to the cognitive improvements observed. The study carried out by Da Silva et al. (2021) revealed that the interval cycling training program was linked to a noteworthy rise in the expression levels of miR-29a-3p, miR-103a-3p, and miR-106a-5p, which in turn was linked to cognitive improvement in males with PD (Da Silva et al. 2021).

### Beneficial actions of physical exercise in multiple sclerosis

Regular exercise is beneficial for individuals with MS, as it enhances physical function, mobility, and manages fatigue (Motl 2020). Exercise programs focusing on aerobic conditioning, strength training, and balance exercises contribute to improved mobility and independence (Motl 2020). Fatigue is a common symptom experienced by MS patients, but regular exercise can help manage it over time. Aerobic exercise, such as walking, cycling, or swimming, improves cardiovascular fitness and lowers the risk of cardiovascular diseases (Motl 2020). Exercise also positively impacts mood and mental well-being, reducing symptoms of depression and anxiety, improving psychological well-being, and enhancing quality of life. Reduced weight-bearing activity and certain medications may increase the risk of osteoporosis and bone fractures in MS patients (Motl 2020). Weight-bearing exercises, resistance training, and balance-promoting activities help maintain bone density and reduce fracture risks. Weight management and metabolic issues can be improved through regular exercise, combined with a healthy diet (Motl 2020). Engaging in fitness regimens provides social interaction and assistance, since MS patients can interact with people who have similar experiences in groups, which lessens feelings of loneliness and builds a sense of belonging.

### Modulation of ncRNAs by physical exercise in multiple sclerosis

MS is a neurodegenerative disorder characterized by inflammation, hypoxia, oxidative stress, and neuroinflammation.

### The role of miRNA by physical exercise in multiple sclerosis

Patients with MS have been shown to have dysregulated expression of several miRNAs, which may be involved in the etiology of the illness. Combined training has been found to restore the expression levels of certain miRNAs, leading to a more balanced and regulated inflammatory response. The researchers conducted experiments using a rat model of MS-like behaviors to investigate the effects of royal jelly consumption and physical activity (Lohrasbi et al. 2022). Royal jelly, known for its various pharmacological properties, was found to have potential therapeutic benefits in halting neurodegeneration. The combination of exercise training and royal jelly supplementation showed a significant impact on the expression of miRNAs, hub genes, and networks associated with MS (Table1) (Lohrasbi et al. 2022). This interaction resulted in improvements in motor function, proinflammatory cytokine levels, and demyelination (Lohrasbi et al. 2022).

MiR-23b is a miRNA that has been implicated in the pathogenesis of MS. When compared to healthy persons, the expression levels of miR-23b in MS patients are changed, indicating a possible function for this gene in the onset and course of the illness. Studies have shown that miR-23b is downregulated in MS patients. This downregulation is associated with increased inflammation and disease severity. MiR-23b is known to have anti-inflammatory properties, and its decreased expression may contribute to the dysregulation of immune responses in MS. MiR-23b has been found to target and regulate several genes involved in immune responses and inflammatory processes. For instance, it has been demonstrated to target the transcription factor NF-κB and interleukin-17 (IL-17), both of which are crucial for the pathophysiology of multiple sclerosis. By downregulating these targets, miR-23b may help suppress inflammation and modulate immune responses in MS. Furthermore, studies have suggested that miR-23b may also be involved in the regulation of oligodendrocyte function and myelination, which are impaired in MS. It has been demonstrated to target genes related to myelin formation and oligodendrocyte differentiation, indicating a possible function for it in fostering MS repair and remyelination processes. On the other hand, miR-155 and miR-326 are two miRNAs that have been extensively studied in the context of MS. These miRNAs play important roles in immune regulation and have been implicated in the pathogenesis of MS. It is known that MS patients have elevated levels of miR-155, especially in immune cells like T and B cells. It is involved in the regulation of various immune processes, including inflammation, immune cell activation, and differentiation. Pro-inflammatory reactions and MS disease activity have been linked to increased expression of miR-155. Studies have shown that miR-155 targets multiple genes involved in immune regulation. It has the ability to alter the expression of important molecules in immune cell signaling pathways as well as pro-inflammatory cytokines, including interferon-gamma (IFN-γ) and tumor necrosis factor-alpha (TNF-α). Dysregulation of miR-155 can disrupt immune homeostasis and contribute to the immune dysregulation observed in MS. MiR-326 is another miRNA that has been implicated in MS. It is upregulated in immune cells, particularly in activated T cells, in MS patients. The control of T cell activity and migration is attributed to miR-326, and deregulation of this gene has been linked to enhanced T cell activation and infiltration into the CNS in MS patients. TLR activation results in the production of proinflammatory and IFN-inducible genes via the Myeloid Differentiation Factor 88 (MyD88) or Toll/IL-1R domain-containing IFN-inducing adapter (TRIF) pathway, which in turn induces downstream signaling pathways. Engaging in regular physical activity may reduce the expression level of TLR-4 in MS patients (Barry et al. 2016). Yousefi Saqqezi et al. (2021) studied how women with relapsing and remitting MS (RRMS) expressed inflammatory markers after completing an eight-week combined exercise training program. The research focuses on Toll-like receptor 4 (TLR-4), miRNAs, and antimicrobial peptides (AMPs), which are known to be implicated in autoimmune disorders. As immunological modulators, AMPs are referred to (Auvynet and Rosenstein 2009). Proinflammatory stimulus can trigger the production of AMPs, which function as a key immune factor in the CNS. IL-17 is one of the cytokines that can cause AMPs to express. In MS patients, there is an overexpression of IL-17 in the brain (Shabgah et al. 2014). TLR-4 activation by AMPs may have pro-inflammatory effects. In this study, twenty-three women with RRMS were randomized to the control group or the combined training group. Twenty-three RRMS women participated in the study and were assigned to either the combined training group or the control group. Twenty-three RRMS women participated in the study and were assigned to either the combined training group or the control group (Yousefi Saqqezi et al. 2021). The expression of anti-inflammatory miRNAs, such as miR-23b and human β-Defensin 2 (hBD-2), was shown to have dropped in both the combined training and control groups; however, the reduction in the combined training group was greater. In the control group, LL-37 expression rose, whereas it stayed unaltered in the combined training group. Both groups had an increase in the expression levels of pro-inflammatory miRNAs, miR-326, TLR-4, and miR-155; however, the combined training group experienced a smaller rise than the control group (Yousefi Saqqezi et al. 2021).

### Beneficial actions of physical exercise in traumatic brain injury

PE is a vital tool for individuals who have experienced traumatic brain injury (TBI). Through the restoration of motor skills and general physical function, as well as the enhancement of muscular strength, coordination, balance, and mobility, it promotes physical rehabilitation (Hornby et al. 2020). Exercise also positively impacts cognitive function, enhancing attention, memory, executive functions, and information processing speed (Zhang et al. 2022). It stimulates neuroplasticity, reorganizing and strengthening neural connections in the brain, leading to cognitive improvements. As well, exercise has antioxidant and anti-inflammatory properties that can facilitate lessen oxidative stress and inflammation in the brain. Exercise may diminish adverse effects of chronic inflammation and oxidative stress, which can hamper neuroplasticity and cognitive efficiency (Daniela et al. 2022).

In addition to improving cognitive function, exercise also has significant benefits for mood and mental health. By promoting the production of endorphins, which are the body’s natural mood-enhancing substances, it lessens the symptoms of stress, anxiety, and sadness. Regular exercise also promotes cardiovascular health, enhancing cardiovascular fitness and reducing the risk of cardiovascular diseases. Adequate sleep is crucial for individuals with TBI, as it helps regulate sleep patterns, improve quality, and promote restorative sleep. Exercise has been shown to reduce insomnia symptoms and promote better sleep in TBI patients.

In the rehabilitation process, exercise helps reclaim functional independence by improving daily living activities. Rehabilitation programs often incorporate exercise as a core component to help individuals regain physical and cognitive abilities (Zhang et al. 2022). Participating in exercise programs also provides opportunities for social engagement and integration, reducing feelings of isolation and improving social skills. Group exercise settings offer a supportive environment and sense of camaraderie.

### Modulation of ncRNAs by physical exercise in traumatic brain injury

A brain injury happens when there is a force or damage to the brain. A variety of incidents, such as slips, vehicle accidents, sporting mishaps, or violent crimes, can result in these injuries. Depending on the severity and degree of the injury, a TBI can be moderate to severe. The effects of TBI can be wide-ranging and vary from person to person. They can impact physical, cognitive, emotional, and behavioral functioning. TBI can cause major cognitive impairments that sometimes interfere with daily functioning. These impairments might include issues with memory, orientation, executive functions, attention, and problem-solving (Dikmen et al. 2009). Nevertheless, the currently available therapies for TBI are still far from providing satisfactory results. There is increasing evidence suggesting that engaging in PE, such as wheel running or treadmill use, after experiencing TBI, can mitigate some of the detrimental effects caused by the injury (Chen et al. 2023). Growing research suggests that PE is the best strategy for improving brain health and cognitive performance. Exercise on demand may be beneficial for treating CNS problems, according to some research. Enhancing neuronal plasticity and cognitive function, decreasing secondary neuronal death, maintaining the rhythmic firing patterns of spinal motoneurons, promoting recovery and brain repair—all of these effects are possible with exercise. Exercise-induced benefits in cognition may be impacted by epigenetic alterations, including histone modifications, DNA methylation, and ncRNAs like miRNAs that regulate these changes (van den Brand et al. 2012).

### The role of miRNA by physical exercise in traumatic brain injury

There is evidence to show that miRNAs may be important for the profound cellular and molecular alterations that occur after TBI, both in the short and long term (Table 1) (Hu et al. 2012, 2015). In the work by Bao et al. (2014), following voluntary exercise training, the expression of miRNAs in the hippocampal regions of mice with TBI was assessed. These changes were assessed using microarray analyses. Spontaneous exercise using a running wheel improved cognitive deficits induced by TBI in the mice (Bao et al. 2014). Moreover, the activity changed the expression of miRNAs in the hippocampal regions of TBI and sham-operated animals. More specifically, the increase in cognitive function that was seen was linked to miR-34a and miR-21 (Bao et al. 2014).

In another study, researchers investigated the potential contribution of miR-21 in the cognitive enhancement observed after mice with TBI engaged in spontaneous RW activity (Hu et al. 2015). The study’s conclusions showed that voluntary RW exercise effectively decreased the increased expression of miR-21 in the hippocampal regions of TBI mice. On the other hand, miR-21 overexpression resulted in a decline in spatial learning and memory retention in TBI mice that had access to a RW. Hippocampal neuron size and branching decreased in tandem with this. In contrast, downregulation of miR-21 reversed these effects (Hu et al. 2015). Based on these results, the researchers concluded that miR-21 is an important molecular factor involved in the neuroprotective effects induced by voluntary RW exercise after TBI. The findings imply that the cognitive recovery seen in TBI mice after exercise may be related to the control of miR-21 expression.

However, evaluating the association between exercise and TBI outcomes requires taking into account pre-TBI activity and its effects on the death rate as well as the recovery of the righting reflex in TBI patients. There is evidence to suggest that engaging in regular exercise before experiencing a TBI may have a positive effect on mortality rates (Miao et al. 2015). Several studies have indicated that individuals who are physically active and have a higher level of fitness prior to sustaining a TBI may have a reduced risk of mortality compared to those who are less active (Miao et al. 2015). Although the precise processes underlying this link are still unclear, it is thought that exercise prior to a TBI may improve physiological resilience, cardiovascular fitness, and general health, all of which may increase the likelihood of survival after a TBI. In the context of TBI, the recovery of the righting reflex is often used as an indicator of neurological recovery and functional improvement. Exercise prior to TBI has been linked to enhanced neuroplasticity and neuroprotective processes, albeit the precise effect of exercise on the recovery of the righting reflex may differ according to the extent and type of the lesion (Miao et al. 2015). These factors may potentially contribute to enhanced recovery of motor functions, including the righting reflex, following TBI. It has been discovered that voluntary exercise prior to TBI alters the expression of miRNA in the damaged mouse cerebral cortex (Miao et al. 2015), and after the brain damage, these modifications in miRNA expression can continue. Using microarray analysis, Miao et al. (2015) observed differential changes in the levels of several miRNAs in the cerebral cortex of TBI mice that had undergone voluntary exercise. Some miRNAs were upregulated, while others were downregulated. Among the groups of sham-non-runners, TBI-non-runners, and TBI-runners, the study found an overexpression of miR-874, miR-92a, and miR-21 and a downregulation of miR-138, let-7c, and miR-124 (Miao et al. 2015). Based on their research, the scientists postulated that miRNAs such as miR-124, let-7c, miR-21, miR-138, miR-92a, and miR-874 may contribute to the preventative and protective effects of voluntary exercise against TBI (Miao et al. 2015). After pre-TBI exercise, the damaged cerebral cortex showed different miRNA expression patterns. These findings might point to the possible role of miRNAs in the underlying processes of exercise-induced neuroprotection and neuroplasticity. These miRNAs may target specific mRNA transcripts, influencing gene expression and subsequent cellular processes related to brain injury and recovery.

### Beneficial actions of physical exercise in spinal cord injury

Regular PE can significantly benefit individuals with spinal cord injury (SCI) by improving physical function, promoting independence, and enhancing muscle strength, endurance, flexibility, and range of motion. It also improves cardiovascular fitness, respiratory function, and overall physical capacity, facilitating daily activities and mobility (Tweedy et al. 2017). Exercise promotes neuroplasticity, allowing the brain and nervous system to adapt and reorganize (Sandrow-Feinberg and Houlé 2015). It stimulates neural pathways, strengthens existing connections, and facilitates the formation of alternative pathways, leading to functional enhancements like motor control, sensory perception, and coordination (Sandrow-Feinberg and Houlé 2015). Joint contractures, osteoporosis, and bone loss can result from SCI (Battaglino et al. 2012); however, weight-bearing activities and resistance training can help preserve bone density, lower the risk of fracture, and enhance joint health. Exercise also prevents muscle atrophy and preserves healthy muscle and connective tissue. Regular aerobic exercise improves cardiovascular fitness, reduces cardiovascular disease risks, regulates blood pressure, enhances circulation, and aids in weight management. Exercise positively affects bladder and bowel function, regulating bowel movements, improving transit time, and reducing the risk of constipation. Exercise also has a major positive impact on physical well-being, lowering stress, anxiety, and depressive symptoms while enhancing self-worth, body image, and general quality of life. Combining regular exercise with a balanced diet can help manage weight, reduce body fat, and improve metabolic parameters. Exercise also helps prevent or manage secondary health conditions associated with SCI, such as cardiovascular diseases and type 2 diabetes (Tweedy et al. 2017).

### Modulation of ncRNAs by physical exercise in spinal cord injury

SCI refers to damage or trauma to the spinal cord resulting in a loss of motor, sensory, and autonomic function below the level of the injury (Alizadeh et al. 2019). It is a complex and potentially devastating condition that can have profound effects on a person’s physical, sensory, and emotional well-being. ncRNAs are essential for both normal function and aberrant alterations in the physiology of spinal cord injuries. Dysregulation of ncRNAs in SCI can lead to altered gene expression related to inflammation, apoptosis, neuronal plasticity, and axonal regeneration (Zhou et al. 2016). It can also disrupt epigenetic mechanisms and influence the formation and composition of the glial scar. Understanding the role of ncRNAs in these processes is crucial for unraveling the underlying mechanisms of SCI and exploring potential therapeutic strategies to promote recovery and regeneration. In order to compare the expression levels of mRNA, miRNA, and lncRNA in SCI rats receiving exercise treatment against those not receiving it, the researchers employed RNA-seq technology (Table1) (Wu et al. 2022).

### The role of lncRNA by physical exercise in spinal cord injury

The analysis of the data reveal various ceRNA networks involving lncRNAs that are potentially involved in molecular pathways contributing to the improvement of recovery in individuals with SCI through exercise. One of these networks had the ceRNAs and the gene Stx7 (Wu et al. 2022). Syntaxin-7 (Stx7) is a protein that plays a crucial role in intracellular membrane fusion events. It belongs to the syntaxin family, which mediates an assortment of cellular functions, including exocytosis and endocytosis, by facilitating the docking and fusing of transport vesicles with target membranes (Wang et al. 1997; Hammarlund et al. 2007). These procedures are essential for neurons to release neurotransmitters. The capacity of these ceRNAs to bind miR-193b-5p and target Stx7 was discovered. It was found that the expression of lnc-MSTRG.12598.1 and MSTRG.18736.1 differed between rats with SCI and rats receiving exercise treatment. Furthermore, it has been proposed that certain lncRNAs, particularly MSTRG.12598.1 and MSTRG.18736.1, might serve as miR-4443 sponges. This implies that these lncRNAs may competitively bind to miR-4443, thereby sequestering it. MiR-4443 has been shown to influence the expression of solute carrier family 9 member A6 (SLC9A6). This suggests that miR-4443 can interact with the mRNA of SLC9A6 and potentially regulate its expression levels. The gene SLC9A6 produces the sodium/hydrogen exchanger 6 (NHE6) protein. The membrane transporter protein NHE6 is involved in preserving ion homeostasis and pH equilibrium in cells. It is primarily found in the endosomes and lysosomes, where it is involved in the regulation of intracellular pH and the transport of ions such as sodium and hydrogen. Alterations in the function or expression of SLC9A6 can impact sensory perception related to touch, pain, temperature, and other somatic sensations (Sharif et al. 2018; Petitjean et al. 2020). It is possible that exercise training will raise SLC9A6/NHE6 levels (Wu et al. 2022), which may then reduce sensory abnormalities after SCI.

NEGR1 (Neuronal Growth Regulator 1) is a gene that encodes a protein involved in neuronal development and function. It is particularly important in processes such as neuronal growth, polarity, and synaptic plasticity (Salluzzo et al. 2023). NEGR1 plays a critical role in nervous system development, contributing to the formation of neural networks and maintaining neuronal health. During early brain development, it facilitates the establishment of connections between neurons, which is essential for creating the complex circuitry necessary for proper brain function. In addition to its developmental roles, NEGR1 is involved in synaptic functions, which are essential for learning and memory processes. By promoting the stability and efficacy of synapses, NEGR1 ensures that neurons can communicate effectively, which is crucial for cognitive tasks. Dysregulation of NEGR1 has been linked to various neurodevelopmental disorders and cognitive impairments, underscoring its importance in maintaining synaptic integrity (Salluzzo et al. 2023). Moreover, NEGR1 exhibits protective effects on neurons under stress, helping to safeguard against cellular damage from factors such as oxidative stress or inflammation. This neuroprotective function suggests that NEGR1 may enhance neuronal resilience, potentially influencing the progression of neurodegenerative diseases. By counteracting stress-induced damage, NEGR1 supports neuronal survival and functionality, making it a key player in strategies aimed at promoting brain health and mitigating neurodegeneration (Salluzzo et al. 2023). The study of NEGR1 not only deepens our understanding of neuronal biology but also holds promise for developing therapeutic interventions targeting neurodegenerative disorders. Research has demonstrated that NEGR1 expression is elevated following SCI, indicating that it plays a role in the damaged spinal cord’s regenerative response (Wang et al. 2022; Poplawski et al. 2018). It is possible that the pathophysiological processes of spinal cord ischemia-reperfusion injury (SCIRI) are influenced by the overexpression of NEGR1. The elevation in NEGR1 expression is enabled by lncRNA MIAT(Myocardial Infarction Associated Transcript), which functions as a rival to miR-150-5p (Wang et al. 2022). MIAT is a lncRNA that is localized in the cell nucleus. MIAT has been directly associated with myocardial infarction (heart attack) and is reported to regulate post-transcriptional processes by acting as a ceRNA, competing with other RNA molecules for shared miRNA binding sites (Zeinelabdeen et al. 2024). MIAT has also been implicated in the pathogenesis of certain NDs, in addition to its well-established role in cardiovascular diseases (Zeinelabdeen et al. 2024). The work by Wang et al. (2022)showed that rats with SCIRI had a better exercise status when MIAT was suppressed utilizing the MIAT knockdown vector (Wang et al. 2022). The results of MIAT knockdown were shown to improve hind limb mobility and locomotor function as measured by the Basso, Beattie & Bresnahan locomotor rating scale (Wang et al. 2022). In ischemic lesions, apoptosis is known to result in tissue loss and damage, and caspase-9 is an essential component of the mitochondrial apoptotic cascade. The results of the investigation showed that the knockdown of MIAT considerably boosted the expression of Bcl-2 in the cells while dramatically lowering the expression levels of Bax, caspase-3, and caspase-9 (Wang et al. 2022). Furthermore, the research showed that in the spinal cord tissue of SCIRI rats, MIAT knockdown led to a decrease in NEGR1 expression (Wang et al. 2022). This suggests that MIAT upregulates NEGR1, potentially through competitive binding with miR-150-5p, as mentioned earlier.

#### The role of miRNA by physical exercise in spinal cord injury

SCI modifies the expression of target genes and changes the expression of miRs implicated in various secondary injury responses, such as, apoptosis, inflammation, and oxidative stress (Liu et al. 2009). Numerous genes in the CNS are regulated by exercise, including those related to cell death, immunological response, and neuronal plasticity, as well as neutrotrophins. A class of proteins known as neurotrophins controls the growth and plasticity of axons in the adult central and peripheral nervous systems as well as synaptic function, neuronal survival, and neurotransmitter release (Keefe et al. 2017). One of the neurotrophins that is most widely distributed and researched in the mammalian brain is BDNF. Following injury, BDNF has a range of neuronal populations in which it promotes growth and provides neuroprotection. BDNF not only protects neurons but also promotes axon sprouting and regeneration in the spinal cord, as well as enhanced axon remyelination (Tuinstra et al. 2012; Zhao et al. 2013). The upregulation of BDNF may contribute to enhanced regeneration. The research carried out by Wu et al. (2022) indicated induction BDNF via exercise. Syndecan-4 (SDC4), part of the Syndecans family, is a transmembrane proteoglycan present on the surfaces of cells. It functions as a receptor for diverse growth factors and molecules in the extracellular matrix (Leblanc et al. 2018; Elfenbein and Simons 2013). The research carried out by Wu et al. (2022) indicates that there is a potential connection between SDC4 and BDNF regulation. According to their research, SDC4 may play a big part in controlling BDNF. The study also reveals that the expression of SDC4 may be modulated by a particular miRNA known as miR-6325, which might affect the protein’s levels (Wu et al. 2022). According to the study’s results, ceRNAs that are produced by exercise may be able to compete with SDC4 for binding to miR-6325 (Zhou et al. 2016). This competition can lead to the sequestration or binding of miR-6325 by the ceRNAs, resulting in a reduction in the available pool of miR-6325 that can interact with SDC4 mRNA.

The PTEN/mTOR pathway is a signaling pathway involved in cell growth, proliferation, and survival. A tumor suppressor gene called PTEN prevents the function of the protein kinase mTOR, which is important in stimulating the development of cells and the creation of proteins. Dysregulation of this pathway can impair neuronal plasticity and hinder recovery after SCI (Liu et al. 2012). Given its involvement in neuroprotection, axonal regeneration, and inflammation modulation, the PTEN/mTOR pathway is considered a potential therapeutic target for SCI. Exercise has been shown to downregulate the expression and activity of PTEN, the negative regulator of the mTOR pathway. By inhibiting PTEN, exercise promotes mTOR activation, which can enhance neuroprotection and facilitate axonal growth and regeneration. In the context of SCI, miRNAs are important regulators of the PTEN/mTOR pathway. It has been discovered that miR-199a-3p and miR-21 are powerful PTEN expression regulators (Liu et al. 2012). Downregulation of PTEN by miR-21and miR-199a-3p results in increased mTOR signaling activity. In rats with SCI, exercise has been demonstrated to control miRNAs that target the PTEN/mTOR pathway (Liu et al. 2012). The researchers conducted experiments on rats that underwent SCI and received cycling exercise as a therapy (Liu et al. 2012). They examined how certain miRNAs expressed themselves in spinal cord tissue at various times following damage, along with their target genes and downstream effectors. They found that miR-21 was expressed more frequently, and miR-199a-3p was expressed less frequently when they exercised (Liu et al. 2012). Significant variations in the expression of their target genes were connected with these changes: mTOR mRNA increased whereas PTEN mRNA declined (Liu et al. 2012). Protein level alterations that were comparable were validated by western blotting. The study also discovered that Rapamycin therapy prevented exercise from causing the phosphorylation of S6, a downstream effector of mTOR, in intermediate grey neurons (Liu et al. 2012). This shows that miRNAs that control the PTEN/mTOR pathway may be involved in at least some of the modulation of exercise-induced plasticity in the damaged spinal cord. These results suggest that SCI-affected neurons may have more capacity for regeneration (Liu et al. 2012). Apoptosis, or programmed cell death, plays a significant role in the secondary injury processes that occur after SCI, contributing to additional neuronal damage. Specific miRNAs have been found to be dysregulated and implicated in the regulation of apoptotic pathways. These miRNAs can either promote or inhibit apoptosis depending on their specific targets. They have the ability to control the production of pro- or anti-apoptotic genes that are engaged in signaling pathways leading to apoptosis. Modulating the expression of these apoptosis-associated miRNAs has shown promising results in experimental models of SCI. Remarkably, in the setting of SCI, exercise treatments have also been seen to affect the expression of miRNAs linked to apoptosis. Exercise has been shown to upregulate miR-21 (Liu et al. 2010), which has anti-apoptotic effects and promotes neuronal survival. In animal models of SCI, increased expression of miR-21 has been linked to improved neuroprotection and functional recovery (Liu et al. 2010). Studies have suggested that upregulation of miR-let-7a may contribute to neuroprotection following SCI. In animal models of SCI, lower neuronal apoptosis and higher neuronal survival have been linked to increased expression of miR-let-7a. MiR-let-7a has also been implicated in axonal regeneration after SCI. It has been demonstrated to target and suppress the expression of genes related to axonal growth inhibition and growth cone collapse, therefore encouraging axonal regeneration and functional recovery. MiR-16 is another miRNA that has been investigated in SCI research. It has been linked to a number of processes important to the pathophysiology of spinal cord injuries and has been discovered to be dysregulated in damaged spinal cords. It has been demonstrated that miR-16 regulates inflammation following SCI. It has the ability to alter the expression of chemokines and pro-inflammatory cytokines, indicating a possible function in immune response modulation and inflammation resolution.

Studies have suggested that miR-16 may contribute to neuronal survival and functional recovery after SCI. In animal models of SCI, enhanced motor function recovery and reduced neuronal death have been linked to increased expression of miR-16. Apoptosis after SCI has been found to be regulated by miR-15b. According to studies, miR-15b expression can affect apoptotic signaling pathways and is dysregulated in spinal cord injuries. In spinal cord injuries, there is increased expression of miR-15b, which has been linked to apoptotic promotion. MiR-15b can target and downregulate anti-apoptotic factors, such as Bcl-2, leading to increased susceptibility to apoptosis in neuronal cells. The dysregulation of miR-15b after SCI suggests its involvement in the amplification of secondary injury processes. By promoting apoptosis, miR-15b may contribute to further neuronal loss and exacerbate the initial injury. By suppressing miR-15b activity, anti-apoptotic factors can be upregulated, promoting neuronal survival and potentially enhancing recovery. After SCI in rats, cycling exercise has been shown to affect the expression of miRNAs linked to apoptosis (Liu et al. 2010). The researchers discovered that while exercise raised levels of miR-21 and lowered levels of miR-15b, SCI boosted expression of miR Let-7a and miR-16 (Liu et al. 2010). Pro- and anti-apoptotic genes, among other target genes, showed similar changes in expression in response to these changes in miRNA expression. Additionally, after exercise treatment, the researchers found a decrease in caspase-7 protein levels and a downregulation of caspase-7 mRNA (Liu et al. 2010). By affecting the expression of many miRNAs and their target genes, our results imply that exercise has positive effects on the control of apoptosis following SCI.

### Effects of exercise on exosomal ncRNAs in neurological diseases

**Fig. 3:**
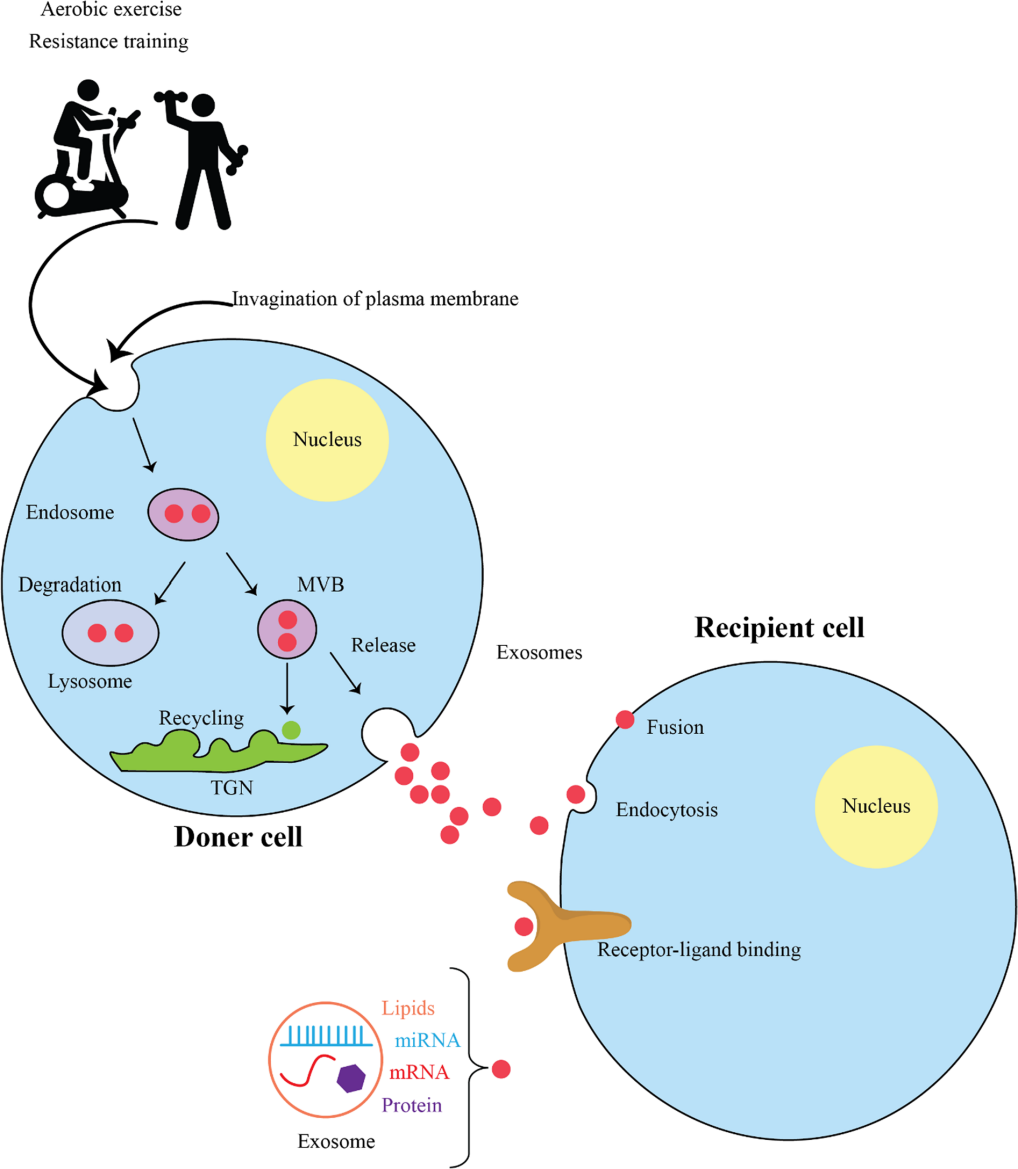
The main process of exosome biogenesis, secretion, and ingestion. The biogenesis of exosomes starts with the formation of early endosomes through endocytosis at the plasma membrane. Then, the early endosomes begin inward budding starting from the membrane to form the endosome lumen, sequestering ncRNAs and other cytoplasmic molecules and eventually converting to intraluminal vesicles. MVBs (multivesicular bodies) are matured from the segregation of the lumen from the plasma membrane leading to molecule accumulation in the intraluminal vesicles, which are also called late endosomes. Generally, MVBs either fuse with the plasma membrane or fuse with the lysosome for degradation. In extracellular space, exosomes are untaken by target cells, mediating by endocytosis, fusion or receptor interaction. As a result, exosome contents are taken into recipient cells and exert biological functions

Early phases of research are being conducted on how exercise affects exosomal ncRNAs in neurological illnesses. Exosomes are small extracellular vesicles that contain various molecules, including ncRNAs, and they play a role in intercellular communication **(**Fig. 3) (Zhang et al. 2015). While limited, some studies have suggested that exercise can influence the content and function of exosomal ncRNAs in the context of neurological diseases. Table 2 provides a summary of the release of exosomes and their contents, as well as the effects of exercise-released exosomes in both cell culture and animal models. Three articles evaluated the impact of exercise on ncRNAs carried by exosomes in neuronal and NDs. Exosomal miR-215-5p expression was observed to be increased in the exercise group by Chen et al. (2022). This increase shows that miR-215-5p levels in exosomes may be elevated by exercise. A particular miRNA called miR-215-5p has the ability to suppress the expression of several genes linked to necroptosis, including BCL2L11, IDH1, and SIRT1. IDH1 is an enzyme involved in cellular metabolism, BCL2L11 is a regulator of programmed cell death, and SIRT1 is a protein involved in various cellular processes, including aging and stress response. Through the suppression of these genes’ expression, miR-215-5p may help prevent neuronal cells from undergoing necroptosis. Additionally, two transcription factors—GATA6 and CEBPB—are identified in the study as potential regulators of miR-215. These transcription factors most likely contribute to the regulation of miR-215-5p expression. According to the study, exercise-induced alterations in GATA6 and CEBPB expression may cause upregulation of miR-215-5p, which would then affect the regulation of exosomal homeostasis (Chen et al. 2022). It has been demonstrated that exercise guards against cognitive decline, and the breakdown of the blood-brain barrier (BBB) is linked to cognitive impairment in AD. Exercise promoted the clearance of Aβ in the brain, improved BBB function, and ameliorated memory impairment in exercised 5XFAD mice (Liang et al. 2023). Exosomes isolated from the brains of exercised mice stimulated cell proliferation and upregulated the expression of PDGFRβ, ZO-1, and claudin-5, which are proteins associated with BBB integrity. These exosomes also showed significant changes in a specific miRNA called miR-532-5p (Liang et al. 2023). A neurological disorder known as mild cognitive impairment (MCI) is characterized by a discernible loss in cognitive function. Between typical aging and dementia, especially AD, MCI is seen as a transitional stage. A study demonstrated a significant statistical link between regular exercise and mild MCI. Findings revealed that individuals who engaged in regular exercise had a decreased likelihood of experiencing MCI compared to those who did not exercise regularly. Conversely, the study found that the plasma exosomes of older MCI patients had considerably higher levels of miR-483-5p and miR-502-5p than those of a control group of people with comparable ages who did not have MCI (Al-Rawaf et al. 2021). By encouraging cellular oxidative stress and apoptosis, expression of miR-483-5p is substantially linked to a more severe cognitive deterioration in MCI patients (Al-Rawaf et al. 2021). Research has demonstrated a favorable association between miR-502-5p and nerve cell viability, death, and inflammation (Chen et al. 2020). Given the potential link between these miRNAs and MCI, it is reasonable to consider that regular exercise could potentially influence the expression or function of these miRNAs.

#### Future directions

Despite emerging evidence, research on the impact of exercise on ncRNAs and exosomal ncRNAs is still limited. Few studies have specifically examined the FITT-VP (Frequency, Intensity, Time, Type, Volume, Progression) parameters of exercise in subjects with NDs. The connection between exercise and ncRNA modulation could offer insights into therapeutic strategies for improving health outcomes in individuals with NDs. More focused research is needed to clarify these relationships and explore the mechanisms involved. Indeed, many studies that investigate the effects of exercise on ncRNAs often fail to control for critical training parameters like volume and progression. This lack of control can hinder the reliability of the findings. Additionally, the tendency to use small sample sizes further limits the generalizability of the results. Addressing these methodological issues is essential for drawing more robust conclusions and developing effective exercise interventions for individuals with neurodegenerative diseases. Future research should prioritize larger, well-controlled studies that comprehensively assess various training parameters. Many studies tend to focus on the acute effects of a single exercise session, assessing immediate serum-level changes in extracellular vesicles. This approach often overlooks the long-term adaptations that can result from consistent exercise regimens. Moreover, stress-related factors can further complicate the interpretation of short-term results, as they may influence the number and content of exosomes. To better understand the impact of exercise on ncRNAs and exosomes, it is crucial that future research includes longitudinal studies that examine the lasting effects of structured exercise programs over time. This will provide a clearer picture of how sustained physical activity can contribute to health improvements in individuals with neurodegenerative diseases.

## Conclusions

Dysregulation of ncRNAs is known to be involved in various aspects of neurodegenerative disease pathogenesis. PE is a non-pharmacological intervention recommended for individuals with NDs, although the precise mechanisms underlying its beneficial effects are not fully understood. While current evidence falls short of establishing causality, it does provide significant biological plausibility to support the potential role of PE in mitigating the dysregulated ncRNAs observed in NDs. This plausibility can generate hypotheses regarding the integrated pathway clusters that may be implicated. PE has the potential to restore an ncRNA profile that supports normal neural stem cell function, brain metabolism, inflammatory status, and other relevant processes. The relationship between exercise parameters and their effects on exosomal cargo levels and ncRNAs expression is complex. Different types of exercise, intensity levels, duration, and frequency can lead to varying biological responses. Different exercise protocols (e.g., aerobic vs. resistance training) can produce distinct effects on exosomal cargo levels and ncRNAs expression profiles. The optimal intensity, duration, and frequency that yield beneficial outcomes is still uncertain. This requires further research to establish clear guidelines. The FITTVP framework influences how exercise affects molecular changes. Each component can alter the body’s response to exercise. Factors like age, fitness level, and genetics may affect how one responds to exercise, complicating the identification of a universal optimal regimen. Ongoing studies are needed to better understand these relationships and develop personalized exercise recommendations. Moreover, investigating the involvement of ncRNAs in the positive effects of PE in NDs may aid in identifying biomarkers and developing new drugs and therapies for these diseases in the future.

## Data Availability

No datasets were generated or analysed during the current study.
